# A systematic review of dose-volume predictors and constraints for late bowel toxicity following pelvic radiotherapy

**DOI:** 10.1186/s13014-019-1262-8

**Published:** 2019-04-03

**Authors:** Rashmi Jadon, Emma Higgins, Louise Hanna, Mererid Evans, Bernadette Coles, John Staffurth

**Affiliations:** 1 Department of Clinical Oncology, Velindre Cancer Centre, Velindre Road, Whitchurch, Cardiff, CF14 2TL UK; 2 Department of Clinical Oncology, Addenbrookes’ Hospital, Box 193, Cambridge, CB2 0QQ UK; 3 Cancer Research Wales Library, Velindre Cancer Centre, Velindre Road, Whitchurch, Cardiff, CF14 2TL UK; 4 School of Medicine, Institute of Cancer and Genetics, Cardiff University, Velindre Cancer Centre, Velindre Road, Whitchurch, Cardiff, CF14 2TL UK

## Abstract

**Background:**

Advanced pelvic radiotherapy techniques aim to reduce late bowel toxicity which can severely impact the lives of pelvic cancer survivors. Although advanced techniques have been largely adopted worldwide, to achieve their aim, knowledge of which dose-volume parameters of which components of bowel predict late bowel toxicity is crucial to make best use of these techniques.

The rectum is an extensively studied organ at risk (OAR), and dose-volume predictors of late toxicity for the rectum are established. However, for other components of bowel, there is a significant paucity of knowledge. The Quantitative Analyses of Normal Tissue Effects in the Clinic (QUANTEC) reviews recommend dose-volume constraints for acute bowel toxicity for peritoneal cavity and bowel loops, although no constraints are recommended for late toxicity, despite its relevance to our increasing number of survivors. This systematic review aims to examine the published literature to seek dose-volume predictors and constraints of late bowel toxicity for OARs (apart from the rectum) for use in clinical practice.

**Methods:**

A systematic literature search was performed using Medline, Embase, Cochrane Library, Web of Science, Cinahl and Pubmed. Studies were screened and included according to specific pre-defined criteria. Included studies were assessed for quality against QUANTEC-defined assessment criteria.

**Results:**

101 studies were screened to find 30 relevant studies. Eight studies related to whole bowel, 11 to small bowel, and 21 to large bowel (including 16 of the anal canal). The anal canal is an important OAR for the development of late toxicity, and we recommend an anal canal Dmean <40Gy as a constraint to reduce late incontinence. For other components of bowel (sigmoid, large bowel, intestinal cavity, bowel loops), although individual studies found statistically significant parameters and constraints these findings were not corroborated in other studies.

**Conclusions:**

The anal canal is an important OAR for the development of late bowel toxicity symptoms. Further validation of the constraints found for other components of bowel is needed. Studies that were more conclusive included those with patient-reported data, where individual symptom scores were assessed rather than an overall score, and those that followed statistical and endpoint criteria as defined by QUANTEC.

## Background

Pelvic radiotherapy is used to treat approximately 17,000 patients per year in the UK with urological, gynaecological and colorectal malignancies [1]. For a significant proportion of these patients, pelvic radiotherapy improves survival outcomes. For others, it reduces the risk of pelvic recurrences, which can both cause distressing symptoms and be difficult to manage.

Although contributing to the cure of many pelvic cancer survivors, pelvic radiotherapy is associated with late toxicity, in particular late bowel toxicity. Serious life-threatening toxicity such as bowel obstruction, fistulae and bleeding requiring transfusion occur in 4–10% of patients 5–10 years after treatment [2]. Furthermore, an important consideration for the growing number of survivors of pelvic cancers is that 50% of patients report late bowel toxicity symptoms which adversely affect their quality of life after pelvic radiotherapy.

Late bowel toxicity is generally attributed to radiation to bowel and rectum and these are considered the organs at risk (OARs). Advanced radiotherapy techniques for pelvic treatments are continually evolving, with the aim of reducing dose to these OARs.

However, to determine whether the dose reductions achieved by these techniques are likely to translate into reduced toxicity for patients requires detailed knowledge of the dose-volume parameters and constraints for these OARs. Once dose-volume constraints are known these can be used to limit the risk of toxicity and potentially allow safe dose escalation with modern delivery techniques.

In 2010 the Quantitative Analysis of Normal Tissue Effects in the Clinic (QUANTEC) review summarised the available dose-volume data for bowel toxicity, with one review focussing on rectum and the other on stomach and bowel. For rectum, an extensively studied OAR, QUANTEC reviewed a large amount of high-quality data and dose-volume constraints for rectum for acute and late toxicity were recommended [3]. These are commonly incorporated into radiotherapy protocols in clinical practice.

However, for bowel there was a relative paucity of data. QUANTEC reviewed data from six papers which examined the dose-volume relationship of bowel with acute bowel toxicity only [4]. For late bowel toxicity, there was no detailed dose-volume relationship analysis described. Studies mentioned were trial data detailing the incidence of late bowel toxicity at the dose-fractionations used within each trial, though no specific dose-volume predictors can be derived from this information.

The QUANTEC reviewers suggest that the constraints identified for acute bowel toxicity may be applied for late bowel toxicity however clarify that “this correlation is not established”. Further, although QUANTEC examined the peritoneal cavity and small bowel loops as OARs, the potential of other bowel components as OARs for bowel toxicity such as sigmoid, duodenum, ileum and anal canal are not detailed.

In a separate paper by Jackson et al., QUANTEC [5] highlighted issues which hinder the development of dose-volume constraints and the pooling of results from different studies, including variations in toxicity endpoint definition, statistical standards, and anatomical definitions of OARs. They recommended several criteria to assess the quality of future dose-volume studies and to facilitate meta-analysis of these studies.

With reduction of late bowel toxicity being a prime aim of advanced pelvic radiotherapy techniques, the lack of clear dose-volume constraints in this setting has been acknowledged and more studies have been reported. This study aims to systematically review published studies examining the dose-volume predictors of all components of bowel (excluding rectum) for late bowel toxicity, including a quality assessment of these studies from criteria derived from QUANTEC.

From this review we aim to determine the clinically useful dose-volume constraints for late bowel toxicity which can guide protocols for advanced pelvic radiotherapy techniques.

## Methods

### Information sources and search strategy

A systematic search was carried out using Medline, Premedline, Embase, Pubmed and Web of Science on 15th October 2013; Updated searches were performed on 10th November 2014, 3rd September 2015 and 1st May 2017 to ensure all new literature was included. Thesaurus and natural language terms around the concepts of “radiotherapy, radiotherapy injuries, side effects, toxicity, intestines bowel, dose, dose fractionation, dose response relationship” were identified for each database. Duplicate references were removed.

### Study selection

Eligible studies were English language studies, involving human adult patients treated for any gastrointestinal, urological or gynaecological malignancies with external beam radiotherapy. Studies correlating the dose-volume relationship of any component of bowel from duodenum to anal canal with late bowel toxicity were included, apart from those focussed on the rectum, given that it has already been extensively studied as an OAR. Late toxicity was defined as more than 3 months from completion of radiotherapy.

Excluded studies were review articles and letters, studies involving brachytherapy only, or stereotactic body radiotherapy. Both full text papers and conference abstracts were considered, however studies with insufficient methodological detail to be able to repeat the method on an independent sample of patients were excluded.

All abstracts were independently screened by two reviewers (RJ, EH) for inclusion. Full papers of abstracts were acquired and further assessed for eligibility, with any discrepancies discussed between the two reviewers. The reference lists of all the included papers were hand-searched for additional references.

### Data extraction and synthesis of results

Bowel can be defined in several different ways and for the purpose of this review studies were divided into those looking at the whole bowel (including bowel loops and peritoneal cavity), small bowel (and its components) and large bowel (and its components). For each included study the number of patients, proportion with the toxicity, tumour site, OAR studied, toxicity definition, treatment details and key findings were tabulated.

Furthermore, the recommendations from QUANTEC [5] on quality of dose-volume studies were reviewed, and those criteria that can be applied to this subject were selected (see Table 1). Each included study was assessed for quality against these statistical and endpoint criteria.

**Table 1:** Statistical and Endpoint Considerations from QUANTEC [5]

Statistical considerations	
1 Basic statistical data provided on incidence of toxicity -Both number of subjects and number of events should be reported -If an estimate of incidence is given the standard error should be supplied	
2 Numerical labeling of response histogram – if into groups eg. quartiles must state number of patients in each quartile	
3 When predictive models are correlated with complications parameter estimates must be stated with their standard error	
4 Complication rates associated with constraints must be reported	
5 “Goodness of fit” to be reported such as Chi-squared	
6 Discriminator statistics reported such as receiver operating characteristic curves	
7 Full organ volumes (rather than partial) should be used -If this is not possible absolute volumes should be used or a standard method of normalization -A clear statement of organ volume definition should be given	
Toxicity Endpoint considerations	
1 Symptom-specific information rather than a portmanteau endpoint (eg. RTOG gr 2) should be used	
2 Consideration that symptoms may be attributed to pre-radiotherapy co-morbidities	
3 Patient-reporting of symptoms may be important	

## Results

**Fig. 1:**
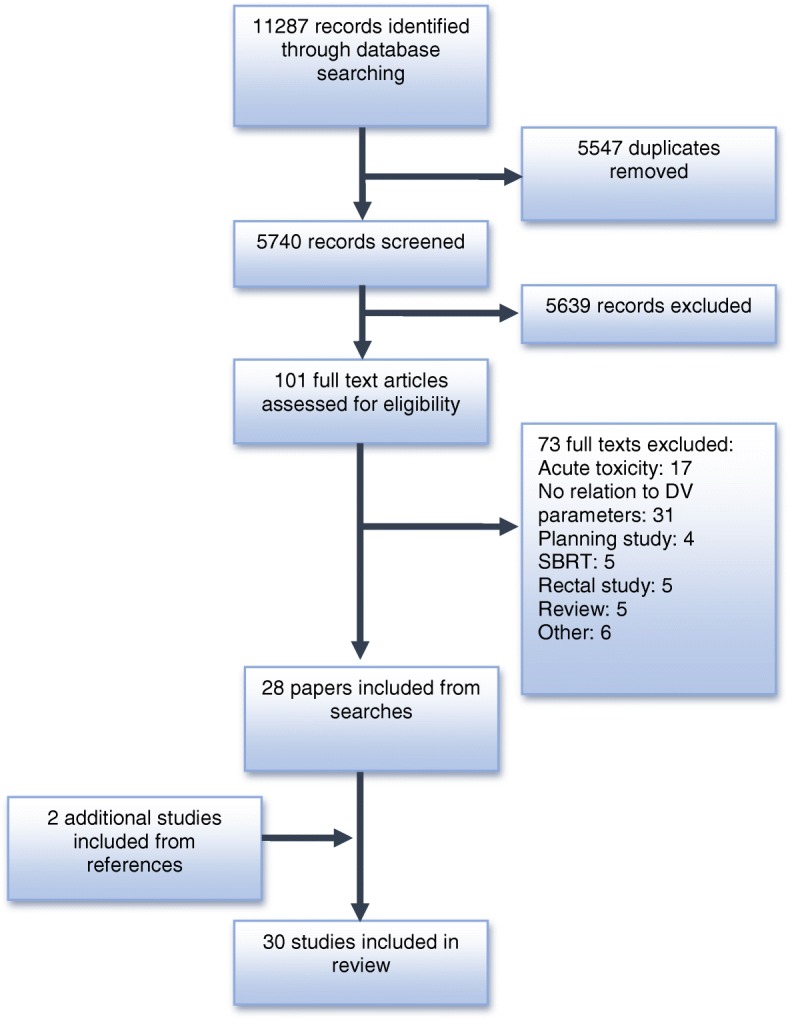
Systematic Search Outcomes

Outcomes of the systematic search are shown in Fig. 1. Overall, 30 studies involving a total of 5126 patients were included as detailed in Table 2. Twenty-one studies included patients with prostate cancer, 6 with gynaecological cancers (cervical and endometrial), and 2 each included bladder and pancreatic cancers. Most studies (*n* = 18) included less than 100 patients, with 9 studies having less than 50 patients included.

**Table 2:** All included studies

Author	Year	Cancer site	No of pts	Pts with tox	OAR studied	Toxicity score used	RT Type	Primary RT Dose (Gy/#)	Pelvic RT dose (Gy/#)	Concurrent chemo use
Adkison [34]	2012	Prostate	53	20	small bowel	CTCAE v3.0	IMRT	70/28	56/28	no
al-Abany [18]	2005	Prostate	65	9	anal sphincter region	Own questionnaire	3D	70.2/39	NS	no
Alsadius [19]	2012	Prostate or prostatic bed	403	51	anal sphincter region	Own questionnaire	3D	70/35	NS	no
Buettner [20]	2012	Prostate	388	57	anal canal	Common grading scheme	3D	64/32 or 74/37	NS	no
Chopra [11]	2014	Cervix (post-op)	71	9	small bowel, large bowel	CTCAE v3.0	IG-MRT (46); 3D (25)	50/25	50/25	63/71 cisplatin
Deville [31]	2010	Prostate	30	2	intestinal cavity	RTOG	IMRT	79.2/44	45/25	no
Deville [7]	2012	Prostatic bed	36	5	intestinal cavity	RTOG	IMRT	70.2/39	45/25	no
Ebert [35]	2015	Prostate	754	Symptom specific	Anal canal	LENT-SOMA	IMRT	66–78/33–38	NS	no
Fokdal [14]	2005	Prostate or bladder	71	Symptom specific	small bowel	LENT-SOMA	Conformal	60/30 (bladder) 69.6/35 (prostate)	48-60Gy bladder; NS for prostate)	no
Fonteyne [17]	2007	Prostate	241	Symptom specific	small bowel, sigmoid	RTOG and “RILIT”	IMRT	74/37–80/40	NS	no
Green [36]	2015	Prostate or prostatic bed	73	10	Intestinal cavity	CTCAE v4.0	IMRT/VMAT	61–79.2	45	no
Guerrero- Urbano [8]	2010	Prostate & Pelvic nodes	79	21	bowel loops	RTOG diarrhoea & LENT SOMA diarrhoea	IMRT	70/35	50/35 or 55/35	no
Huang [15]	2011	Pancreas	46	8	duodenum	CTCAE v4.0	3D or IMRT	42/15	42/15; 36/15; 38/19	Gemcitabine; 18 pts. erlotinib in addition
Isohashi [10]	2013	Cervix (post-op)	97	16	peritoneal cavity, small and large bowel	RTOG/EORTC	2D or 3D	50/25	50/25	All nedaplatin
Kelly [13]	2013	Pancreas	106	20	duodenum	CTCAE v4.0	3D or IMRT	50.4/28 (78pts); 57.5–75.4 in 28–39# (28pts)	50.4/28 (78pts); 57.5–75.4 in 28–39# (28pts)	Gemcitabine 5-FUor capecitabine +/− cetuximab or erlotinib
Author	Year	Cancer site	No of pts	Pts with tox	OAR studied	Toxicity score used	RT Type	RT Dose (Gy/#)	Pelvic RT dose (Gy/#)	Concurrent chemo use
Koper [25]	2004	Prostate	266	141	anal canal	RTOG (simplified) Symptom questionnaire	3D or 2D	66/33	NS	no
Lind [12]	2016	Cervical or Endometrium	519	63	Anal sphincter, small bowel, sigmoid	Own questionnaire (defecation into clothing without forewarning)	2D or 3D	40–46 (endometrium) or 55–70 (cervix)	NS	Not stated
Mavroidis [27]	2005	Prostate	65	Symptom specific	anal sphincter	Own questionnaire	3D	70.2/39	NS	no
Mcdonald [9]	2015	Bladder	47	10	bowel loops	RTOG	3D	64/32	64/32	21 received 5-FU/MMC
Mouttet-Audouard [6]	2015	Cervical	37	8	Small bowel [defined as peritoneal cavity], sigmoid	CTCAE v4.0	IMRT (tomotherapy)	60/28	50/28	Cisplatin
Peeters [21]	2006	Prostate	641	146	Anal wall	RTOG/EORTC plus 5 specified symptoms	3D (41 pts. had IMRT boost)	68/34 or 78/39	NS	no
Peeters [28]	2006	Prostate	368	32	Anal wall	Incontinence (no specific questionnaire)	3D (22 pts. had boost)	68/34 or 78/39	NS	no
Poorvu [16]	2013	Cervix or Endometrium (+ PA nodes)	46	3	peritoneal cavity, small bowel, duodenal segments	CTCAE v4.0	IMRT	45/25 (22pts); PAN boost 50–65 (33pts)	45/25 & PAN boost 50–65 (33 pts)	24 received cisplatin
Smeenk [26]	2012	Prostate	48	21	Anal sphincter muscles	Presence of frequency, urgency and incontinence	3D (*n* = 43, IMRT (*n* = 5)	67.5/27 or 70/28	NS	no
Smeenk [22]	2012	Prostate	36	23	Anal wall	Late RILIT score: urgency, incontinence, frequency	3D	67.5/27 or 70/28	NS	no
Taussky [37]	2003	Prostate	73	unclear	anal canal	UCLA, FACT-P and EORTC QLQ-PR25	3D	66.6–72/ 37–40	NS	no
Thor [29]	2015	Prostate	212	Symptom specific	Anal sphincter	Own questionnaire with 19 descriptors for 4 symptoms	3D	70-78Gy	NS	no
Verma [30]	2014	Cervix & Endometrium	105	9	duodenum	RTOG and endoscopic findings	IMRT	45–50 (60-66Gy boost)	45–50 (60–66 boost)	58 pts. platinum agents
Vordermark [23]	2003	Prostate or prostatic bed	44	14% severe incontinence	anal canal	10 question continence questionnaire	3D	58–72/29–36	NS	No
Yeoh [24]	2016	Prostate	106	72%	Anal wall	LENT-SOMA total score	3D	66–74.4/ 33–4	NS	no

*Abbreviations*: *Pts* Patients, *OAR* Organs at risk, *RT* Radiotherapy, *Gy* Gray, *#* Fraction, *NS* Not stated, *CTCAE* Common terminology criteria for adverse events, *RTOG* Radiation therapy oncology group, *LENT-SOMA* Late Effects of Normal Tissue – Subjective Objective Management Analytical, *RILIT* Radiation induced late intestinal toxicity, *EORTC* European Organisation for Research and Treatment of Cancer, *IG-IMRT* Image-guided intensity modulated radiotherapy, *IMRT* Intensity modulated radiotherapy, *VMAT* Volumetric modulated arc therapy, *PA nodes* Para-aortic nodes, *pts* Patients

Table 3 details studies for whole and small bowel, and Table 4 those for large bowel. In each table the final two columns indicate the quality assessment criteria of statistical and endpoint considerations as defined in Table 1. If a specific criterion is met its number is noted in the column.

**Table 3:** Whole bowel and small bowel studies – significant findings and quality assessment

						Quality Assessment	
Author	OAR studied	OAR defined	Toxicity definition	Pts with toxicity	Significant findings	Statistical criteria met (1–7)	Endpoint criteria met (1–3)
Deville [7]	Intestinal cavity	Intestinal cavity below L4–5	RTOG Gr ≥ 1	5/36 (14%)	Toxicity associated with total volume & V20. No constraints specified.	1,7 (n/a: 2–6)	None
Mouttet-Audouard [6]	“Small bowel” [outlined as abdominal cavity hence included in this section]	Entire abdominal cavity including all possible organ locations to iliac crests or D12/L1	CTCAE v4.0 Gr1–3 –diarrhoea or “whole digestive toxicity” (diarrhoea, gastritis, bleeding, pain, incontinence)	8/37 (21.6%) 17/37 (46%)	Larger volumes of bowel receiving 10–30Gy associated with diarrhoea & whole digestive toxicity. (No constraints specified) “Whole digestive toxicity” associated with many parameters including D20%-D95%.	1, 7 (n/a 2–6)	2
Green [36]	Intestinal cavity	Not stated	CTCAE v4.0	9 (12%)	No dose-volume relationship found.	1 (n/a 2–6)	2
Deville [31]	Intestinal cavity	Large & small bowel below L4–5	RTOG Gr ≥ 2	2/30 (6%)	No dose-volume relationship found	1,7 (n/a 2–6)	None
Isohashi [10]	Peritoneal cavity	Volume surrounding small bowel loops to edge of peritoneum excluding bladder & rectum	RTOG/EORTC Gr ≥ 2	16/97 (16.5%)	No dose-volume relationship found	1,7 (n/a 2–6)	2
Poorvu [16]	1. Peritoneum 2. Peritoneum + Colon	1. Possible location of small bowel excluding solid organs & retroperitoneal structures. 2. Peritoneum (as above) plus asc & desc colon	CTCAE v4.0 Gr > 3	3/46 (6.5%)	No dose-volume relationship found	1, 7 (n/a 2–6)	2
Guerrero-Urbano [8]	Bowel loops	Loops from recto-sigmoid junction to 2 cm above PTV	RTOG Gr ≥ 2 diarrhoea; LENT-SOMA consistency & frequency- worst grade	21/79 (26%) RTOG diarrhoea; ≥gr2 6/79 (7.6%)	V40, V45, V60 and bowel volume of > 450 cc had both higher RTOG & LENTSOMA diarrhoea. Constraints suggested: V40 < 124 cc, V45 < 71 cc, V60 < 0.5 cc for RTOG<gr 2	1,7 (n/a 2–3)	1
McDonald [9]	Bowel loops	Loops from recto-sigmoid junction to 2 cm above PTV	RTOG Gr ≥ 1	7/47 (14.9%) gr1; 3/47 (6.4%) gr2	Constraints for < 25% ≥ gr2 toxicity: V30 < 178 cc;V35 < 163 cc;V40 < 151 cc;V45 < 139c; V50 < 127 cc; V55 < 115 cc; V60 < 98 cc V65 < 40 cc	1,4,7 (n/a 2–3)	2
Chopra [11]	Small bowel	2 cm above target, individual small bowel loops (unclear how differentiated from large bowel)	CTCAE v3.0 Gr3+	9/71 (12.6%)	V15 associated with ≥gr3 toxicity. Recommend V15 < 275 cc, V30 < 190 cc, V40 < 150 cc reduces Gr3 toxicity from 23.6 to 5.6%.	1, 4, 6 (n/a 2,3)	2
Isohashi [10]	Small bowel	Bowel loops remaining after exclusion of large bowel loops	RTOG/EORTC Gr ≥ 2	16/97 (16.5%)	V40 best predictor of late toxicity; Recommend V40 < 340 ml to reduce toxicity from 46.2 to 8.7%	1,4,6,7 (n/a 2,3)	2
Lind [12]	Small bowel	All visible small bowel in small pelvic cavity to caudal part of sacroiliac joints	Defecation into clothing without warning > 1 in last 6 months	63/519 (12.1%)	Mean dose>50Gy to small bowel or sigmoid or anal sphincter region associated with symptom (findings for individual organs not clarified)	1, 7 (n/a 2,3)	1,2,3
Adkison [34]	Small bowel	Not clearly defined	CTCAE v3.0 Gr1 and Gr2	Gr1 16/53 (30%); Gr2 4/53 (8%)	No dose-volume relationship with V30-V60 small bowel	1 (n/a 2–6)	None
Fokdal [14]	Small bowel	Opacified & unopacified small intestine loops (outer contour & contents) from 1st slice to minor pelvis	LENT-SOMA G1–4	Symptom specific	No dose-volume relationship found	1,7 (n/a 2–6)	1,2,3
Fonteyne [17]	Small bowel	Not clearly defined	RTOG and “RILIT” Gr1 & Gr2	Gr1 112/241 (46%), Gr2 32/241(13%)	No dose-volume relationship found	1, (n/a 2–6)	1,2
Poorvu [16]	Small bowel	Opacified & non-opacified small bowel loops	CTCAE v4.0 Gr3+	3/46 (6.5%)	No dose-volume relationship found	1,7 (n/a 2–6)	2
Huang [15]	Duodenum	Duodenal bulb to ligament of Treitz	CTCAE v4.0 Gr ≥ 3	8/46 (17.4%)	With a V25 > 45% toxicity rates increase from 8 to 48%	1,4,6,7 (n/a 2,3)	2
Kelly [13]	Duodenum	Gastric pylorus until end of duodenum 3 cm past midline	CTCAE v4.0 Gr ≥ 2	20/106 (18.9%)	With a V55 > 1 cc toxicity rates increase from 9 to 47%	1,4,6,7 (n/a 2,3)	2
Verma [30]	Duodenum	From gastric outlet through transverse portion of duodenum (ascending portion excluded)	RTOG, all grades	9 /105 (8.6%)	With a V55 > 15 cc toxicity rates increase from 7.4 to 48.6%	1,4,6,7 (n/a 2,3)	2
Poorvu [16]	Duodenal segments	D1 segment: bulblike shape & origin beyond gastric pylorus. Transitions between 2nd & 3rd segments was lateral border of IVC; Between 3rd & 4th was medial border of aorta	CTCAE v4.0 Gr ≥ 3	3/46 (6.5%)	No dose-volume relationship found with duodenum	1,7 (n/a 2–6)	2

*Abbreviations*: *Pts* Patients, *OAR* Organs at risk, *RT* Radiotherapy, *Gr* Grade, *CTCAE* Common terminology criteria for adverse events, *RTOG* Radiation therapy oncology group, *LENT-SOMA* Late Effects of Normal Tissue – Subjective Objective Management Analytical, *RILIT* Radiation induced late intestinal toxicity, *EORTC* European Organisation for Research and Treatment of Cancer, *Vx* Volume receiving x Gy, *AUC* Area under curve

**Table 4:** Large Bowel studies - details and quality assessment

						Quality Assessment	
Author	OAR studied	OAR defined	Toxicity definition	Pts with toxicity	Significant findings	Statistical considerations met (1–7)	Endpoint considerations met (1–3)
Chopra [11]	Large bowel	2 cm above target, individual loops of large bowel (unclear how differentiated from small bowel)	CTCAE v3.0 Gr ≥ 3	9/71 (12.6%)	V15 associated with ≥gr 3 toxicity. Constraints: V15 < 250 cc, V30 < 100 cc, V40 < 90 cc to reduce toxicity from 26.7 to 5.4%	1, 4, 6 (n/a 2,3)	2
Isohashi [10]	Large bowel	Single loop continuing from end of sigmoid to ascending colon	RTOG/EORTC, Gr ≥ 2	16/97 (16.5%)	No constraint found for large bowel	1,7 (n/a 2–6)	2
Fonteyne [17]	Sigmoid colon	Where rectum sweeps anteriorly to one slice above aortic bifurcation	RTOG and “RILIT” Gr 1 and 2	Gr 1112/241 (46%), Gr 2 32/241 (13%).	V40 associated with gr1 diarrhoea & blood loss. Constraints: V40 < 10%, V30 < 16% to avoid gr1–2 diarrhoea	1, 7 (n/a 3)	1,2
Mouttet-Audouard [6]	Sigmoid colon	Anterior curvature of sigmoid colon to anterior abdominal wall	CTCAE v4.0 Gr1–3 diarrhoea and “whole digestive toxicity”	8/37 (21.6%) diarrhoea; 17/37 (46%) (whole tox)	‘Whole late digestive toxicity’ associated with V30–40. No specific constraints.	1,7 (n/a 2–6)	1,2
Lind [12]	Sigmoid colon	From where rectum deviates from its mid- position to where it turns cranially in left abdomen connecting to colon descendens	Defecation into clothing without warning > 1 in last 6 months	63/519 (12.1%)	Mean dose>50Gy to small bowel or sigmoid or anal sphincter region associated with symptom (findings for individual organs not clarified)	1, 7 (n/a 2–6)	1,2,3
al-Abany [18]	Anal sphincter region	Caudal 3 cm of the rectum from anal verge (including filling)	Own questionnaire; Faecal leakage >2X/week	9/65 (13.8%) faecal leakage	Increased risk with mean dose of 45-55Gy. Constraints: V35 < 60%, V40 < 40% associated with no risk of faecal leakage.	1, 7 (n/a 2,3)	1,2,3
Alsadius [19]	Anal sphincter region	Caudal part of large bowel, from end of rectal ampulla where bowel no longer had visible content or air.	Own questionnaire; Faecal leakage >once per month	51/403 (12.7%) faecal leakage	Dmean<40Gy reduces risk from 17 to 4%.	1,2,4,7 (n/a 3)	1,2,3
Fokdal [14]	Anal canal	Outer contour of the structure extending from anal verge 2 cm cranially	LENT SOMA score	Urge: 27/71 (38%); Incontinence: 21/71 (30%)	Urgency related to Dmed> 33.8: increases toxicity 31 to 47% Incontinence related to Dmax> 53.8 increases 14 to 44%	1,2,4,5,7 (n/a 3)	1,2,3
Vordermark [23]	Anal canal	Anal verge to the section below visible rectal lumen, corresponding to the upper border of the levator ani muscle	“Solid soiling” (Severe incontinence) Own continence questionnaire	6/44 (14%)	Severe incontinence - associated with Dmin (23.1Gy) - related to portals extending 2 mm below ischial tuberosities (compared with 5 mm above)	1, 7 (n/a 2–3)	1,2,3
Koper [25]	Anal canal	Caudal 3 cm of the intestine	RTOG gr1 + 2; Plus symptom questionnaire.	141/248 (57%)	D90% (=54.9Gy) to associated with ≥ gr1 rectal toxicity	1, 7 (n/a 2–6)	2,3
Taussky [37]	Anal canal	Most distal 2-3 cm of rectum	10 questions from UCLA-PCI, FACT-P & EORTC QLQ -PR25	Unclear	no relation with anal canal DVH found	7 (N/a: 2–3)	3
Buettner [20]	Anal canal	Caudal 3 cm of rectum	Common grading scheme; subjective sphincter control at highest grade	57/388 (14.7%)	DSH data: Toxicity correlated with dose to anal surface: lateral extent 53Gy > 56%. DVH data: Dmean 47Gy to anal sphincter volume correlated with sphincter toxicity. Constraints: Dmean<30Gy.NTCP modeling to LKB model TD50 = 120, m = 0.42.	1,3,6,7 (n/a 2)	1,2,3
Peeters [21]	Anal wall	Wall of caudal 3 cm of anorectum (method described)	RTOG/EORTC ≥ gr 2 and ≥ gr 3 Plus incontinence pad use>2x/wk.	≥gr 2165/641 (25.7%) ≥ gr 3 27/641 4.2%	Dmean increase from 19Gy to 52Gy increased gr2 toxicity: 16 to 31%. V65 & Dmean most significant for incontinence. Dmean increase by 33Gy increased incontinence by 12%	1,2,4,6,7 (n/a 3)	1,2
Mavroidis [27]	Anal sphincter region	Musculaure layer around the rectal aperture, 3 cm caudal from anal verge	Own questionnaire	faecal leakage 19/65 (29%); blood/mucus 22/65 (34%)	Relative seriality NTCP model of anal sphincter for incontinence, blood/mucus. Parameters for incontinence: *D*50 = 70.2Gy, *γ* = 1.22, *s* = 0.35. Parameters for blood/mucus: *D*50 = 74.0Gy, *γ* = 0.75, *s* ≈ 0	1, 3, 5, 6, 7 (n/a 2)	1,3
Peeters [28]	Anal canal wall	Wall of caudal 3 cm of anorectum (method described)	Incontinence requiring pad use>2x/wk.;	32/368 (7%)	NTCP LKB model of incontinence with anal wall dose. Parameters found were *n* = 7.48; TD50 = 105; m = 0.46	1,3,4,5,6,7 (n/a: 2)	1,3
Smeenk [26]	Anal sphincter muscles	Individual muscles defined (Internal anal sphincter (IAS), external anal sphincter (EAS), puborectalis & levator ani)	Frequency, Urgency, Incontinence	21/48 (44%)	For complication <5% Dmean<30Gy to IAS; <10Gy to EAS, < 50Gy to puborectalis, <40Gy to levator ani	1, 4,5 (n/a 2)	1,2,3
Smeenk [22]	Anal wall	Continuation of rectal wall from anal verge to slice below lowest slice with a rectal balloon	Frequency, urgency, incontinence	39% frequency, 31% urgency, 31% incontinence	For urgency: Anal wall Dmean<38Gy risk < 15%, >38Gy risk is 62%	1,4,7 (n/a 2,3,5,6)	1,3
Lind [12]	Anal sphincter region	Inner muscle layer of the sphincter up to anal verge	Defecation into clothing without warning > 1 in last 6 months	63/519 (12.1%)	Mean dose>50Gy to small bowel or sigmoid or anal sphincter region associated with this symptom (findings for individual organs not clarified)	1, 7 (n/a 2,3)	1,2,3
Yeoh [24]	Anal wall	From anorectal junction (not clearly defined)	LENT-SOMA total score	72%	Anal wall V40 > 65% associated with chronic toxicity.	1,5 (n/a 2,3)	2,3
Thor [29]	Anal sphincter	Anal canal, inner and outer sphincter (not clearly defined)	Questionnaire of 19 questions in 4 domains: pain urgency, mucus & incontinence.	Specific to each of 19 question	5 LKB models proposed for anal sphincter doses. Low anal sphincter dose associated with faecal leakage and pain. High anal sphincter dose associated with leakage.	1,3,6 (n/a 2)	1,2,3
Ebert [35]	Anal Canal	Caudal 3 cm of anorectum	LENT-SOMA – 8 symptoms	Specific to each symptom	Bleeding associated with >40Gy, proctitis with 36-63Gy, frequency with 8-85Gy, urgency and tenesmus with 5-34Gy to anal canal.	1,5,7 (n/a 2)	1,2,3

*Abbreviations*: *Pts* Patients, *OAR* Organs at risk, *RT* Radiotherapy, *Gr* Grade, *CTCAE* Common terminology criteria for adverse events, *RTOG* Radiation therapy oncology group, *LENT-SOMA* Late Effects of Normal Tissue – Subjective Objective Management Analytical, *RILIT* Radiation induced late intestinal toxicity, *EORTC* European Organisation for Research and Treatment of Cancer, *Vx* Volume receiving x Gy, *AUC* Area under curve, *Dmean* Mean dose, *Dmax* Maximal dose, *DVH* Dose volume histogram, *DSH* Dose surface histogram, *NTCP* Normal Tissue Complication Probability, *LKB* Lyman Kutcher Burman

### Whole bowel

Eight papers (including 445 patients) examined the dose-volume relationship of whole bowel either using bowel loops or intestinal/peritoneal cavity as detailed in Table 3.

### Peritoneal cavity

Late bowel toxicity was associated with low doses to the peritoneal cavity (V10–30Gy) in 2 studies. Mouttett-Audouard et al. [6] found, in 37 cervical cancer patients, an association between “bigger volumes” of bowel receiving 10–30Gy and grade 1–3 Common Terminology Criteria Adverse Events (CTCAE) toxicity, although specific cut-offs were not reported. Deville et al. [7] found that peritoneal cavity volume and V20 were both associated with grade 1 Radiation Therapy Oncology Group (RTOG) toxicity. Again no constraints were derived.

### Bowel loops

Two studies [8, 9] investigated bowel loops as an OAR for late toxicity, both with an identical definition of bowel loops. Guerrero-Urbano et al., in 79 patients who had their prostate and pelvic nodes treated, found V40, V45 and V60 bowel loops to be predictive of late grade 2 RTOG-graded diarrhoea. They suggested constraints of V40 < 124 cc, V45 < 71 cc and V60 < 0.5 cc to reduce grade 2 RTOG toxicity, although no complication rates associated with these constraints are detailed. McDonald et al. in their study of 47 bladder cancer patients suggested constraints to reduce the risk of grade ≥ 2 RTOG toxicity to less than 25% (V30 < 178 cc; V40 < 151 cc; V45 < 139 cc; V60 < 98 cc and V65 < 40 cc), although it must be noted that only 3 patients within this study had grade 2 toxicity.

### Small bowel and its components

Eleven studies (including 1401 patients) were included in this section, with 6 studies examining small bowel and 4 examining the duodenum, as detailed in Table 3. No papers investigating the ileum or jejunum were found.

### Small bowel

2 of 6 studies found positive correlations with late bowel toxicities and small bowel volume parameters in cervical cancer patients. However, the positive parameters were different between the studies, with Isohashi et al. [10] recommending a V40 < 340 cc, and Chopra et al. [11] recommending a V15 < 275 cc. Lind et al. [12] found that a mean small bowel dose >50Gy was of significance, however could not clarify whether toxicity was linked specifically to small bowel, sigmoid or anal sphincter dose, making these results difficult to interpret.

### Duodenum

3 of 4 studies found positive correlations between dose-volume parameters and duodenal toxicity. Two studies found V55 to be an important predictor, though with differing constraints. Kelly et al.*,* in 106 pancreatic patients recommending a V55 < 1 cc [13] and Verma et al. in 105 gynaecological patients recommending a V55 < 15 cc [14]. Huang et al. [15] found V25 to be the significant predictor for pancreatic cancer patients treated with concurrent gemcitabine; with a V25 < 45% toxicity rates were 8%, above this constraint toxicity was 48%. Investigation of individual duodenal segments did not reveal any positive findings [16].

### Large bowel and its components

21 studies (including 5006 patients) were included in this section (see Table 4), with 2 examining large bowel, 3 examining sigmoid and 16 studies examining the anal canal/sphincter region.

### Large bowel and sigmoid Colon

Chopra et al. [11] found on multivariate analysis that V15 of large bowel was associated with grade 3 CTCAE toxicity (*p* < 0.03), and recommended with the use of the constraints-V15 < 250 cc, V30 < 100 cc and V40 < 90 cc grade 3 toxicity could reduce from 26.7 to 5.4%.

For the sigmoid colon Fonteyne et al. [17] found in 241 prostate patients that sigmoid V40 was associated with grade 1 diarrhoea and blood loss; they recommended V40 < 10% and V30 < 16% to avoid grade 1–2 diarrhoea. Mouttet-Aldouard et al. [6] also found sigmoid V30–40Gy to be significantly correlated (*p* < 0.006) with “digestive toxicity” although no specific cut-offs were defined.

### Anal canal

15 of 16 studies had positive findings relating dose-volume parameters and Normal Tissue Complication Probability (NTCP) models of the anal canal/sphincter to late toxicity. Most defined the anal canal as the distal 3 cm of rectum.

#### Dmean

5 studies [18–22] found Dmean anal canal or anal sphincter region to be most predictive of toxicity, 4 of faecal incontinence and 1 of faecal urgency, as summarised in Table 5. There was relative consistency in the recommended Dmean constraints between 40-47Gy, despite the OARs being defined slightly differently.

**Table 5:** Anal canal Dmean results

Study	No of pts	OAR	Endpoint	Dmean (in EQD2) constraint	Risk of endpoint below this constraint	Risk of endpoint above this constraint
Al-albany [18]	65	Anal sphincter region	Incontinence >2X/week	43.2	8%	52%
Alsadius [19]	403	Anal canal	Incontinence > 1x/month	40	5.2%	21%
Buettner [20]	388	Anal sphincter region	Incontinence: moderate/severe (gr2)	47, though <30Gy ideal	5% (approx; read from graph)	
Smeenk [22]	36	Anal canal wall	Urgency present	41.8	15%	62%
Peeters [21]	641	Anal canal wall	Incontinence requiring pad >2x/week	No constraint specified	16% at 19Gy	31% at 52Gy

#### Other dose volume histogram (DVH) and dose-surface histogram (DSH) parameters

Many other DVH parameters of the anal canal were found to be important, including Dmin [23], Dmax, Dmedian [14], V40 [24], V65 [21] and V90% dose [25]; these were all in individual findings with little corroboration between studies. Vordermark et al. also found that the treatment field border was important, with those with a lower border 2 mm below ischial tuberosities more likely to have severe incontinence compared with 5 mm above the ischial tuberosities.

Buettner et al. [20] also examined incontinence using dose surface maps (DSM) for the anal canal. They found the mean dose to the anal surface and the lateral extent of the DSM to be most correlated with subjective sphincter toxicity. They recommend 45Gy for surface-based mean-dose to the anal canal to reduce toxicity.

#### Anal sphincter muscles

Smeenk et al. [26] related dose to individual sphincter muscles to urgency, frequency and incontinence. To reduce urgency and incontinence to below 5% they recommended a mean dose <30Gy to internal anal sphincter, <10Gy to the external sphincter, < 50Gy to puborectalis and < 40Gy to the levator ani muscles.

#### Normal tissue complication probability (NTCP) modeling

Four studies detailed NTCP models for the anal canal [20, 27–29], three of fitting data to a Lyman-Kutcher-Burman (LKB) model. Buettner et al. identified mean-dose anal canal parameters related to grade 2 RTOG toxicity, and Peeters et al. looked at anal wall parameters in relation to faecal incontinence, as detailed in Table 4. Peeters et al. further modified their model to incorporate a previous history of abdominal surgery and found this improved the model fit, suggesting a decreased radiation tolerance for patients with this risk factor. Thor et al. [29] proposed LKB models for pain, mucus and faecal leakage, although their findings are difficult to use practically as within their study they use data from two different centres, where each toxicity is defined differently between centres. Mavroidis et al. [27] modelled dose to the anal sphincter region for ‘faecal leakage’ and ‘blood or phlegm’ in stools using the relative seriality NTCP model. They recommended a reduction in the biologically effective uniform dose (EUD) to anal sphincter < 40–45Gy may significantly reduce toxicity.

### Quality assessment

#### Statistical criteria

Most studies provided information on basic statistical data (29/30) and gave clear definitions of OARs (24/30). Constraints were derived in 16 papers, with associated complication rates stated in 12 papers. Goodness-of-fit was reported in 6 studies, with discriminator statistics reported in 10 papers. For the 4 papers with NTCP models all provided parameter estimates with standard error.

#### Endpoint criteria

Overall toxicity grades rather than individual symptoms were assessed in 13 of 30 studies, with patient-reported outcomes used in 14 studies (13 of which were studies of the anal canal). 21 of the studies looked at co-morbidity to assess its contribution to late toxicity and this was taken into account in multivariate analyses if thought to be associated.

## Discussion

We have systematically reviewed the currently published literature on dose-volume constraints for late bowel toxicity after pelvic radiotherapy, excluding the rectum. We identified 30 studies including 5136 patients. A key finding was consistent dose-volume constraints defined for the anal canal from five studies. For whole bowel loops, small bowel, duodenum, large bowel and sigmoid dose-volume constraints were derived in individual studies, however there was limited validation of these findings in other studies examining the same component of bowel.

Of all the components of bowel studied, most data were available in the 16 studies examining the anal canal or anal sphincter region, and these studies were most conclusive. Statistical and endpoint measures recommended by QUANTEC were met much more frequently in these studies, data of which originated mainly from prostate clinical trials. Fifteen of these sixteen studies used individual symptoms reported by patients rather than an overall toxicity score.

These studies clearly indicate a relationship between dose-volume parameters to the anal canal and faecal incontinence. Dmean was the most significant parameter in five different studies, with a range of doses between 40-47Gy found. From the available data we recommend a constraint Dmean of <40Gy (in 2Gy fractions) to the anal canal to be included in clinical protocols in order to limit late bowel toxicity, in particular faecal incontinence.

For other components of bowel, the evidence was far less conclusive, as the findings of single studies were not corroborated with others. Possible reasons could be differences in the endpoint studied (i.e toxicity, grade and clinician versus patient-reporting) and differences in the definition of the OARs. Furthermore different studies derive constraints with different aims, with some using constraints to lower the risk of toxicity to a certain level eg. to 5% or to 20%, and others attempt to derive constraints with the aim of no toxicity at all.

For example when considering constraints for bowel loops, both Guerrero-Urbano et al. [8] and McDonald et al. [9] derived constraints for V40, V45 and V60 associated with late bowel toxicity. However, the constraints in these studies differed, with one suggesting V40 < 124 cc, V45 < 71 cc and V60 < 0.5 cc, and the other recommending V40 < 151 cc, V45 < 139 cc and V60 < 98 cc, despite the same definition of bowel loops, and use of RTOG scoring. Reasons for these differences could be due to differences in endpoint definition, with one study looking specifically at ≥ grade 2 RTOG diarrhoea, with the other looking at overall RTOG toxicity ≥ grade 1. McDonald et al. determined constraints aiming to reduce the risk of ≥grade 1 toxicity specifically to less than 25%, whereas Guerrero-Urbano et al. found constraints with the aim of reducing ≥grade 2 toxicity, but the level to which this aims to reduce the risk of toxicity is unclear.

A similar lack of consistency was seen for studies focussed on duodenum [13, 30], large bowel [10] and sigmoid colon [6, 17] making it difficult to further recommend constraints for these OARs. Future validation of the published constraints using independent data sets from patients using the same toxicity endpoints, same OAR definitions and the same aim in terms of toxicity reduction, would be a useful next step to improve knowledge on this subject.

Many studies found no correlation with OAR dose parameters and late bowel toxicity at all. This lack of findings could be due to a variety of methodological reasons – many of the studies were underpowered with only a very small incidence of the defined toxicity, making it difficult to determine the likely predictors of these toxicities in only a handful of patients. Many studies have not collected baseline data and presume the presence of bowel symptoms post-radiotherapy is treatment related, when in fact these symptoms may have been pre-existing, or due to a separate bowel pathology. Further a known issue within the pelvis, is that of organ motion of bowel and its subsection, and the use of a single CT scan to define a highly mobile structure may not be an appropriate approach.

Aside from methodology the reason for lack of positive findings may be in fact that particular OARs may genuinely not have any influence on late toxicity, and rather than dose-volume predictors, other considerations such as inherent radiosensitivity of individual patients, may be the main predictors of toxicity.

For acute bowel toxicity QUANTEC have suggested two constraints: V45 < 195 cc for peritoneal cavity, and V15 < 120 cc for small bowel loops. The QUANTEC authors suggest these constraints may be applicable for late bowel toxicity. Some consistency is seen to QUANTEC recommendations within this review with Chopra et al. [11] finding V15 small bowel loops to be important on multivariate analysis, although their recommended constraint was much higher at V15 < 275 cc.

For peritoneal cavity, the findings of the studies reviewed do not corroborate with QUANTEC. Three studies found no correlation of peritoneal cavity doses with late toxicity, and the two positive studies found that in fact lower doses to peritoneal cavity of V20 and V10–30 [6, 31] were predictive of late toxicity. It would be important to validate the significance of these low doses in terms of late toxicity given the increased use of volumetric arc therapy (VMAT) techniques in recent years, where lower dose bath to a larger area of normal tissue is seen, the significance of which is currently not understood.

Strengths of this systematic review are the broad inclusivity of the search, with the studies included having patients with different tumour types, radiotherapy techniques, fractionations, and concurrent treatments. A similar approach was used in key papers such as the Emami et al. data [32], as well as the QUANTEC papers [3, 4], where bowel constraints were sought from studies with gynaecological, rectal, prostate and pancreatic cancers. A potential limitation of this is that some of these treatment factors may influence late toxcity (e.g. use of concurrent systemic agents, or hypofractionation). Although it is expected that individual authors may account for these factors statistically this may not have always been done and may explain partly the inconsistent results found.

Despite attempting to be as inclusive as possible we may have missed those studies not in English, and from grey literature currently unpublished. Studies involving SBRT were excluded given the questionable validity of the linear quadratic model with extreme hypofractionation thus making radiobiological comparisons difficult [33].

Quality assessment based on the QUANTEC-defined criteria added much value to this review, highlighting that many researchers do not report or consider the endpoint or statistical criteria, and further that those that do adhere to these criteria appear to have more conclusive findings.

## Conclusions

We recommend the use of Dmean to the anal canal of <40Gy in pelvic radiotherapy protocols as a constraint to reduce the development of late bowel toxicity, in particular faecal incontinence.

Other important organs at risk to consider are whole bowel loops, small bowel, duodenum, large bowel and sigmoid colon and constraints for these OARS are noted in this review. However, clear recommendations for these organs cannot be made, due to lack of correlation between studies. Validation of the constraints found within this systematic review for these OARS with independent data sets would be an important next step. If validated these constraints could be used clinically in prospective patients, and also as a relevant benchmark to assess the likely impact of advanced radiotherapy techniques on late toxicity. Future studies should consider the quality criteria recommended by QUANTEC.

## Abbreviations

Cc: Cubic centimetres
CTCAE: Common Terminology Criteria Adverse Events
Dmean: Mean Dose
DSH: Dose Surface Histogram
DVH: Dose Volume Histogram
EUD: Equivalent Uniform Dose
Gy: Gray
LKB: Lyman-Kutcher Burman
OAR: Organ at Risk
QUANTEC: Quantitative Analysis of Normal Tissue Effects in the Clinic
RTOG: Radiation Therapy Oncology Group
VMAT: Volumetric Modulated Arc Therapy
Vx Gy: Volume receiving x Gray
